# Correction: Stage-specific IFN-induced and IFN gene expression reveal convergence of type I and type II IFN and highlight their role in both acute and chronic stage of pathogenic SIV infection

**DOI:** 10.1371/journal.pone.0193629

**Published:** 2018-02-23

**Authors:** Nadia Echebli, Nicolas Tchitchek, Stéphanie Dupuy, Timothée Bruel, Caroline Passaes, Nathalie Bosquet, Roger Le Grand, Christine Bourgeois, Benoit Favier, Rémi Cheynier, Olivier Lambotte, Bruno Vaslin

The fifth author’s name is spelled incorrectly. The correct name is Caroline Passaes. The correct citation is: Echebli N, Tchitchek N, Dupuy S, Bruel T, Passaes C, Bosquet N, et al. (2018) Stage-specific IFN-induced and IFN gene expression reveal convergence of type I and type II IFN and highlight their role in both acute and chronic stage of pathogenic SIV infection. PLoS ONE 13(1): e0190334. https://doi.org/10.1371/journal.pone.0190334
