# Supplementary material for: Stage-specific IFN-induced and IFN gene expression reveal convergence of type I and type II IFN and highlight their role in both acute and chronic stage of pathogenic SIV infection
Source: PLoS One. 2018 Jan 11;13(1):e0190334. doi: 10.1371/journal.pone.0190334 (PMC5764266; doi:10.1371/journal.pone.0190334)
Supplement: S1 Fig — (PDF) [file pone.0190334.s001.pdf]

A

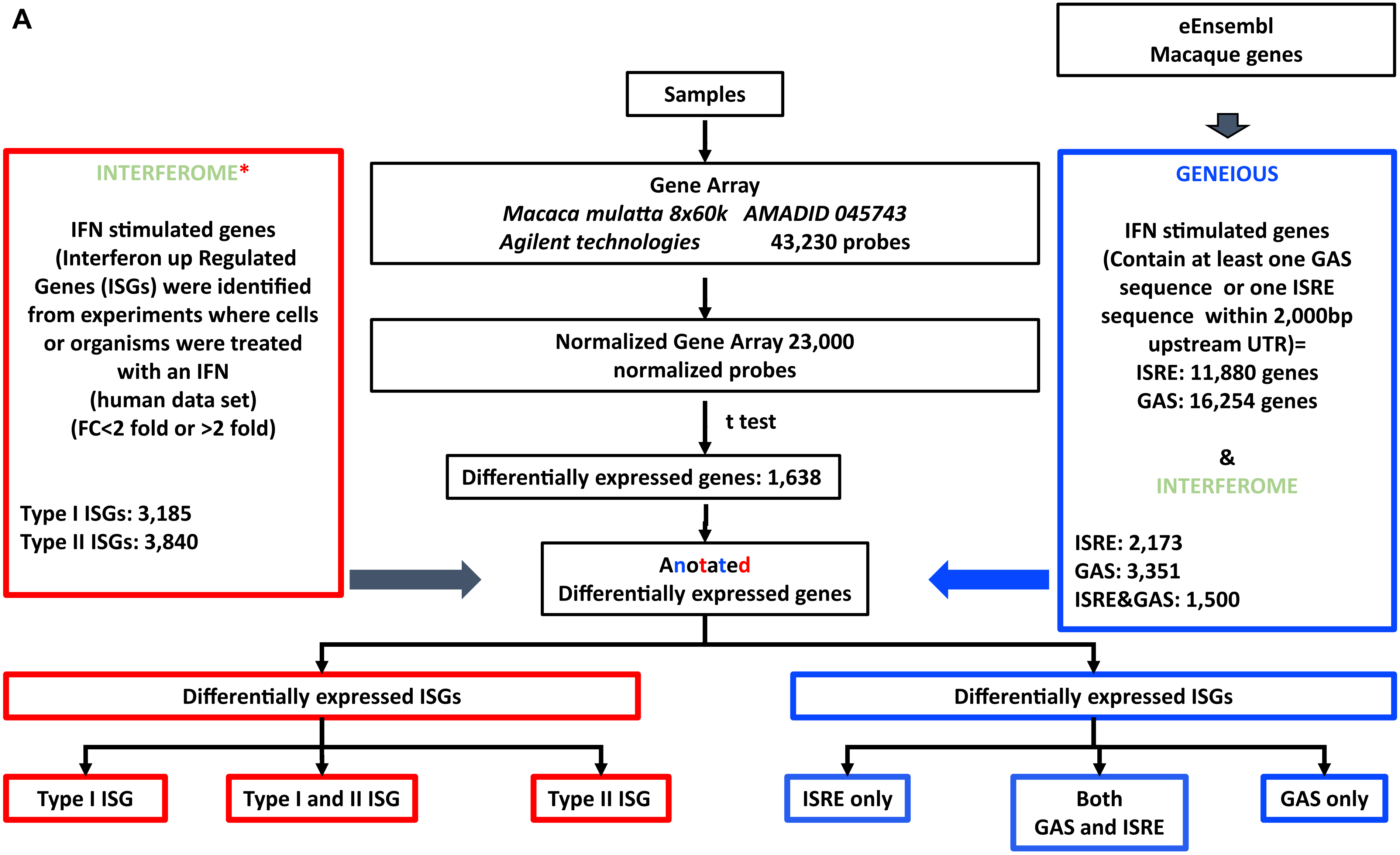

B

ISRE-1 AAAnnGAAAC
ISRE-2 AAAnnGAATG
ISRE-3 GAAnnGAAAC
ISRE-4 GAAnnGAAAG

GAS-1 TTCCnnnAAA
GAS-2 TTACnnnAAA
GAS-3 TTCCnnnAAG
GAS-4 TTACnnnAAG

Supplementary Figure 1: ISG annotations. Differentially expressed interferon-induced genes were annotated using two approaches: the Interferome database helped to identify and classify genes depending on their induction by either type I IFN (α, β), type II IFN (γ), or both IFN types. In addition, Geneious software was used to annotate ISGs depending on the presence of either GAS, ISRE, or both sequence motifs within the 2,000 bp upstream of the 5'UTR of each gene. (**A**) Schematic diagram of annotations. (**B**) ISRE and GAS sequences.
