# Supplementary material for: Stage-specific IFN-induced and IFN gene expression reveal convergence of type I and type II IFN and highlight their role in both acute and chronic stage of pathogenic SIV infection
Source: PLoS One. 2018 Jan 11;13(1):e0190334. doi: 10.1371/journal.pone.0190334 (PMC5764266; doi:10.1371/journal.pone.0190334)
Supplement: S2 Fig — (PDF) [file pone.0190334.s002.pdf]

Supplementary Figure 2

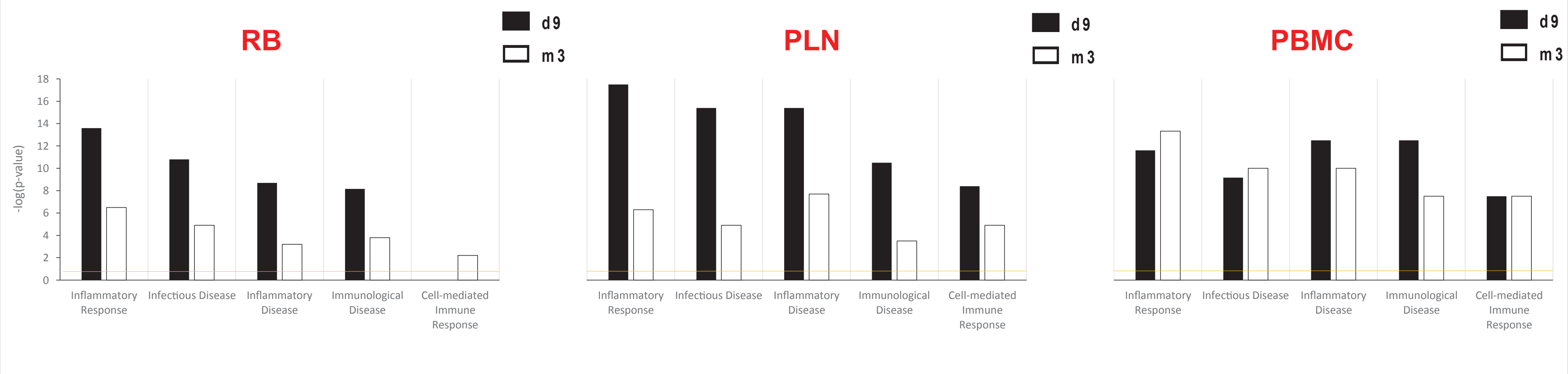

Supplementary Figure 2: Functional enrichment of differentially expressed genes. IPA enrichment analysis was performed on all differentially expressed genes (non-parametric t-test $p < 0.05$) independently of their FC. The –log (p-values) of most significant functions are plotted in a histogram format for both D9 p.i. and M3 p.i., in each tissue including RBs, PLNs, and PBMCs.
