# Supplementary material for: Stage-specific IFN-induced and IFN gene expression reveal convergence of type I and type II IFN and highlight their role in both acute and chronic stage of pathogenic SIV infection
Source: PLoS One. 2018 Jan 11;13(1):e0190334. doi: 10.1371/journal.pone.0190334 (PMC5764266; doi:10.1371/journal.pone.0190334)
Supplement: S3 Fig — (PDF) [file pone.0190334.s003.pdf]

Supplementary Figure 3

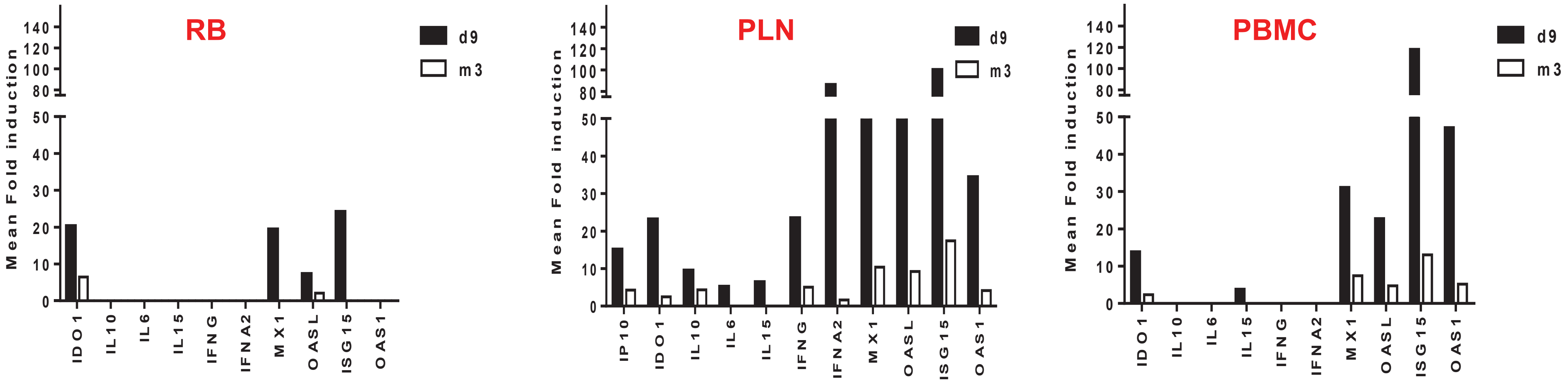

Supplementary Figure 3: Comparison of the extent of IFNs and selected ISG expression in both phases of infection in RM, PLNs, and PBMCs. The mean FC induction shows that both IFNs and ISGs were more consistently induced in PLNs than in PBMCs or RM.
