# Supplementary material for: Stage-specific IFN-induced and IFN gene expression reveal convergence of type I and type II IFN and highlight their role in both acute and chronic stage of pathogenic SIV infection
Source: PLoS One. 2018 Jan 11;13(1):e0190334. doi: 10.1371/journal.pone.0190334 (PMC5764266; doi:10.1371/journal.pone.0190334)
Supplement: S4 Fig — (PDF) [file pone.0190334.s004.pdf]

Supplementary Figure 4

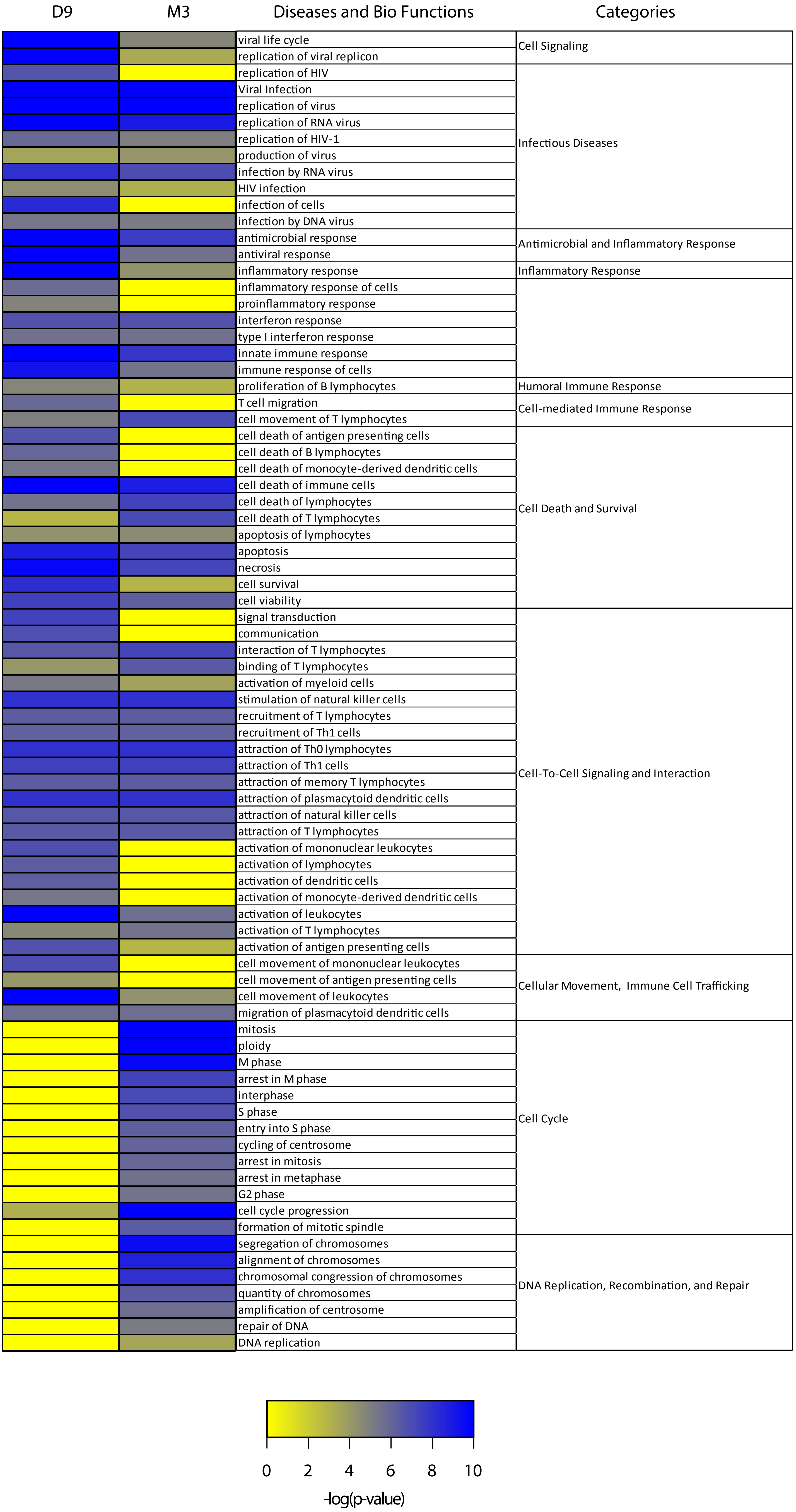

Supplementary Figure 4: The main IPA functions associated with D9-ISGs and M3-ISGs. IPA enrichment analysis was performed on all ISGs. The –log (p-values) of globally significant functions are plotted in heatmap format for both D9 and M3 p.i.
