# Supplementary material for: Stage-specific IFN-induced and IFN gene expression reveal convergence of type I and type II IFN and highlight their role in both acute and chronic stage of pathogenic SIV infection
Source: PLoS One. 2018 Jan 11;13(1):e0190334. doi: 10.1371/journal.pone.0190334 (PMC5764266; doi:10.1371/journal.pone.0190334)
Supplement: S5 Fig — (PDF) [file pone.0190334.s005.pdf]

Supplementary Figure 5

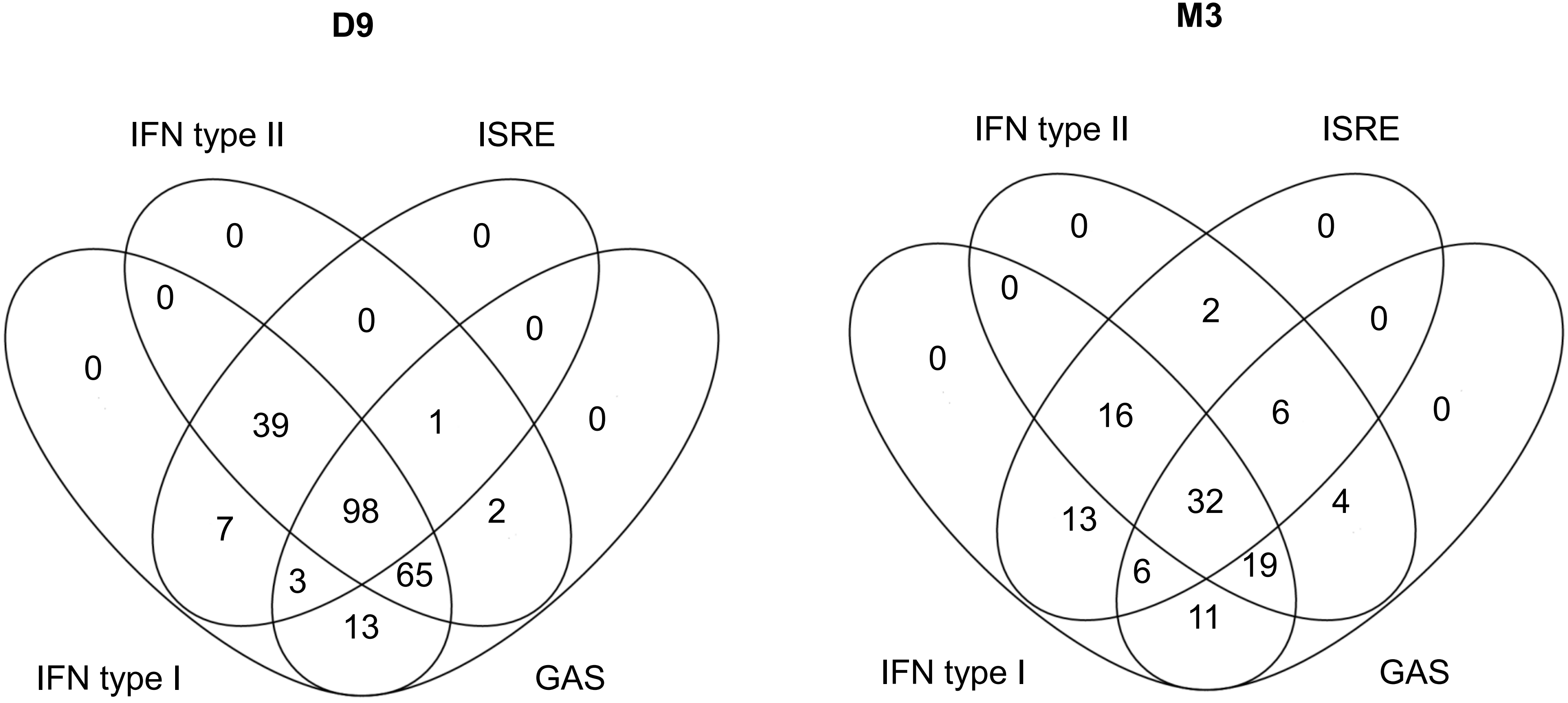

Supplementary Figure 5: Overlap between Interferome annotation and GAS/ISRE annotation of differentially induced ISGs. Venn diagram of differentially expressed ISGs, showing the number of genes in each annotation subset for D9 or M3 p.i.
